# Bilateral Popliteal Vein Thrombosis, Acute Pulmonary Embolism and Mild COVID-19

**DOI:** 10.7759/cureus.11213

**Published:** 2020-10-28

**Authors:** Kulachanya Suwanwongse, Nehad Shabarek

**Affiliations:** 1 Internal Medicine, Lincoln Medical Center, New York, USA

**Keywords:** covid, pulmonary embolism, dvt, acute deep vein thrombosis

## Abstract

In 2020, the coronavirus disease 2019 (COVID-19) has become a global health disaster. Patients with COVID-19 have variable clinical features and unpredictable prognoses; the infectious complication may occur in many organs, causing a broad spectrum of symptoms and severity. Pulmonary embolism (PE) is a fatal urgent complication, which may occur following a severe infection. While the pathogenesis of PE in COVID-19 remains uncertain, it has mainly occurred in patients with severe disease. PE, as an initial presentation of COVID-19 in a patient with mild diseases, is rare and understudied. Here, we describe a young woman with mild COVID-19 illness and no significant risk factors for PE, except obesity, but had developed bilateral popliteal vein thrombosis and submassive PE. Our case emphasizes that thrombotic complications can occur in any COVID-19 patients regardless of the disease severity, questioning the role of preventive anticoagulants in mild COVID-19 cases with certain risk factors.

## Introduction

Novel coronavirus disease 2019 (COVID-19) has now become a fatal pandemic affecting millions worldwide. The causative agent, severe acute respiratory syndrome coronavirus 2 (SARS-CoV-2), is the third identified human pathogenic β-coronavirus, following severe acute respiratory syndrome coronavirus (SARS-CoV) and middle east respiratory syndrome coronavirus (MERS-CoV) [1]. Like SARS-CoV, SARS-CoV-2 enters host cells by binding to angiotensin‐converting enzyme 2 (ACE-2) receptors causing a spectrum of diseases varying from flu-like illnesses to lethal pneumonia [1,2]. For an unclear reason, thrombosis is the major coagulopathy related to SARS-CoV, and SARS-CoV-2, while bleeding diathesis is more common among MERS-CoV [3]. Pulmonary embolism (PE) is a common fatal thrombotic complication associated with severe COVID-19 [4,5]. However, there is limited evidence regarding PE in mild COVID-19 patients. Herein, we share our institutional experience - a young, healthy woman who developed submassive PE and bilateral popliteal vein thrombosis following mild COVID-19.

## Case presentation

An obese 31-year-old woman came to our hospital because of worsening dyspnea. She reported progressive exertional dyspnea associated with chest pain for two days but denied having a fever or any other pulmonary symptoms. The patient endorsed painless swelling of the right leg four days prior. She denied previous medical diseases and was not taking any medication or oral contraceptive pills. None of her family members had a history of vascular thrombosis. The patient denied recent immobilization, including long-distance travel. Her vital signs were: heart rate 110 bpm, blood pressure 135/77 mmHg, body temperature 36.8^0^C, respiratory rate 30 per minute with oxygen saturation of 92% on room air. Her blood test results were demonstrated in table 1; briefly, she had mild leukocytosis, lymphocytopenia, and elevated D-dimer and pro-BNP.

**Table 1:** Initial blood results of the patient

Labs	Patient’s results	Reference values	unit
WBC	11,830	4,080-10,800	cells/mcl
Neutrophils	76.9	44-77	%
Lymphocytes	16.4	20-45	%
Platelets	262,000	150,000-450,000	/mcl
Creatinine	0.67	0.50-0.90	mg/dl
D-dimer	1,853	<230	ng/ml
ESR	100	0-15	mm/hr
Ferritin	125	15-150	ng/ml
Pro-calcitonin	0.09	< 0.08	ng/ml
LDH	369	135-214	U/L
INR	1.2	<1.3	mg/dl
PT	14.2	10.0-13.0	seconds
PTT	27.9	25.0-35.3	seconds
Fibrinogen	391	205-398	mg/dl
Trop T	<0.01	<0.01	ng/ml
Pro-BNP	9,007	<124	pg/ml
CPK	49	<120	U/L
Lactate	1.7	0.5-2.2	mmol/L

**Figure 1:**
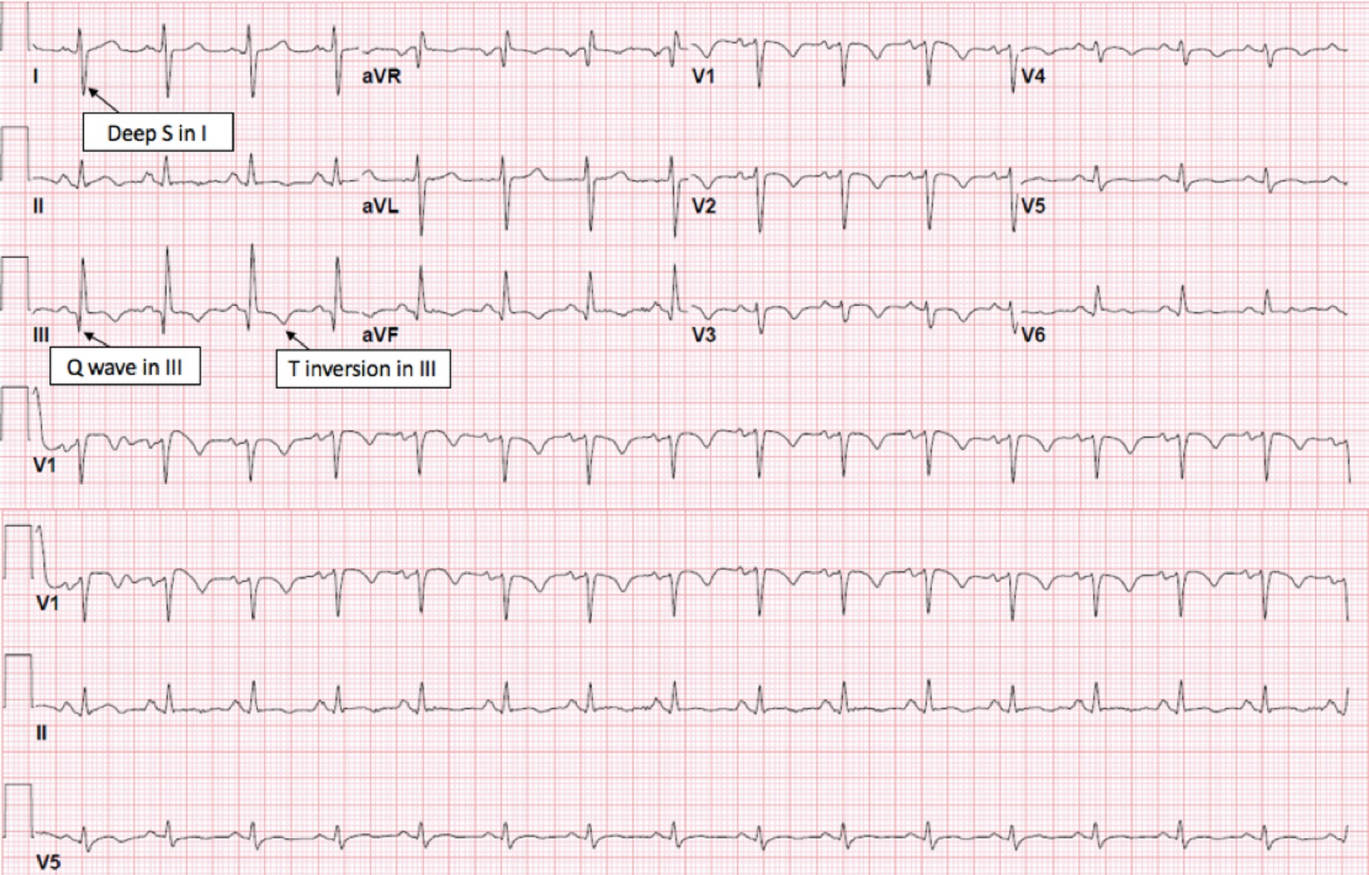
EKG illustrated normal sinus rhythm with S1Q3T3 and anterolateral T wave inversion

**Figure 2:**
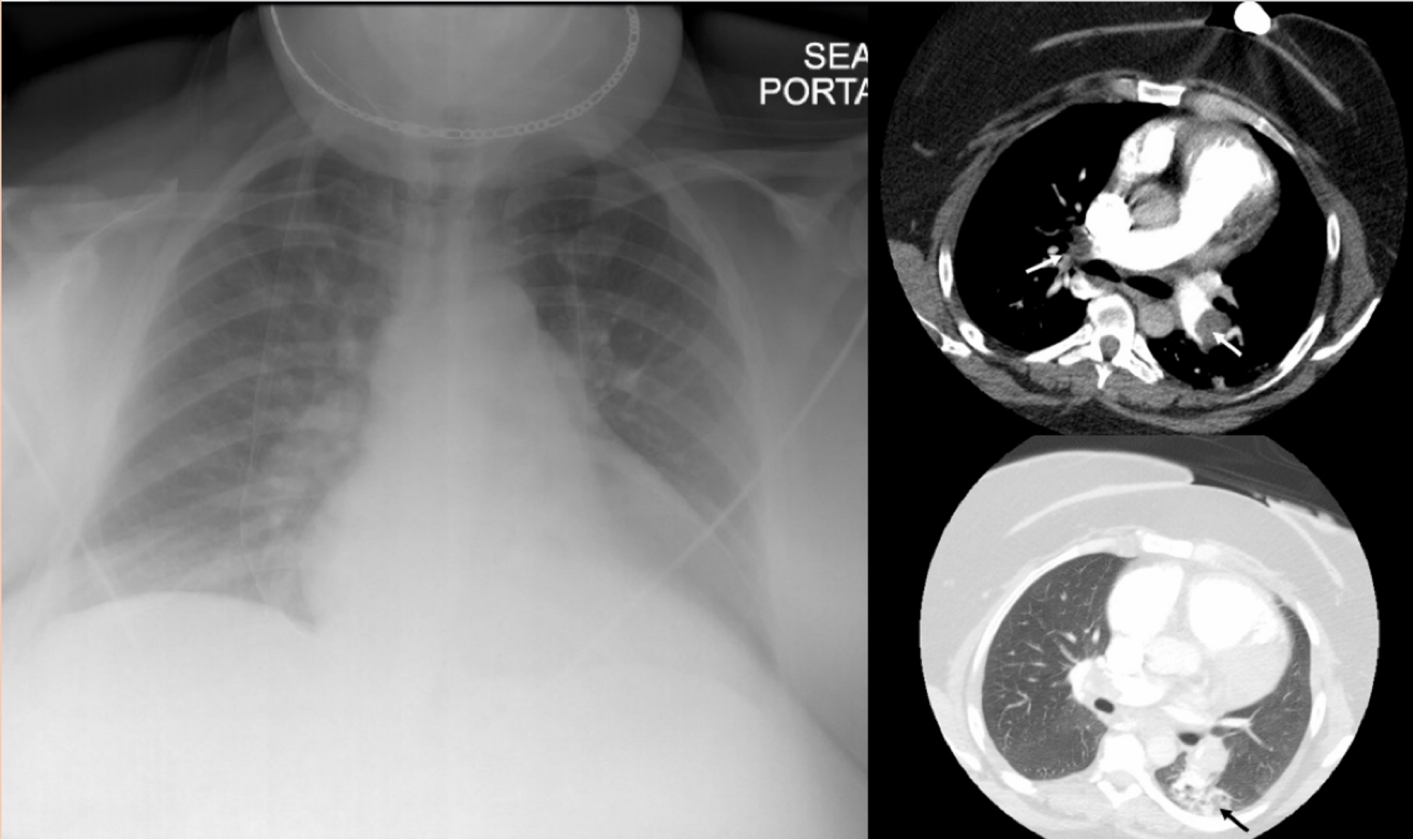
CXR revealed no significant infiltration (left), CT chest angiogram revealed thrombus in bilateral pulmonary arteries (white arrow) and peripheral ground-glass appearance (black arrow)

Her electrocardiogram (EKG) illustrated normal sinus rhythm, S1Q3T3 pattern, and anterolateral T wave inversion (right ventricular strain) (Figure 1). Chest X-ray (CXR) had no significant infiltration (Figure 2, left). Computed tomography (CT) pulmonary angiography showed prominent bilateral pulmonary emboli and peripheral alveolar infiltrations (Figure 2, right). Doppler vascular sonography of bilateral lower extremities found occlusive intravenous thrombosis of bilateral popliteal veins.

She was started on a heparin drip, admitted to the medical intensive care unit, and received catheter-directed thrombolysis for the treatment of PE. She did not receive any specific COVID-19 medication. On admission day 4, her dyspnea and hypoxia were resolved, and she was discharged on a 6-month course of oral anticoagulant.

## Discussion

Previous scoping review estimated that 20% of critically ill COVID-19 patients developed venous thromboembolism (VTE); the development of VTE was also found to adversely impact patients’ outcomes and was associated with high COVID-19 mortality [6]. However, there is a lack of data regarding VTE in mild COVID-19. Ullah et al. recently reported a 59-year-old woman with multiple comorbidities admitted due to COVID-19 pneumonia and hypoxemia requiring supplemental oxygen therapy. Before planned discharge, the patient suddenly developed worsening hypoxemia and was found to have massive PE; she received therapeutic enoxaparin with clinical improvement and was discharged home [7]. We described a young woman presented with worsening dyspnea and was found to have mild COVID-19 and submassive PE. 

**Figure 3:**
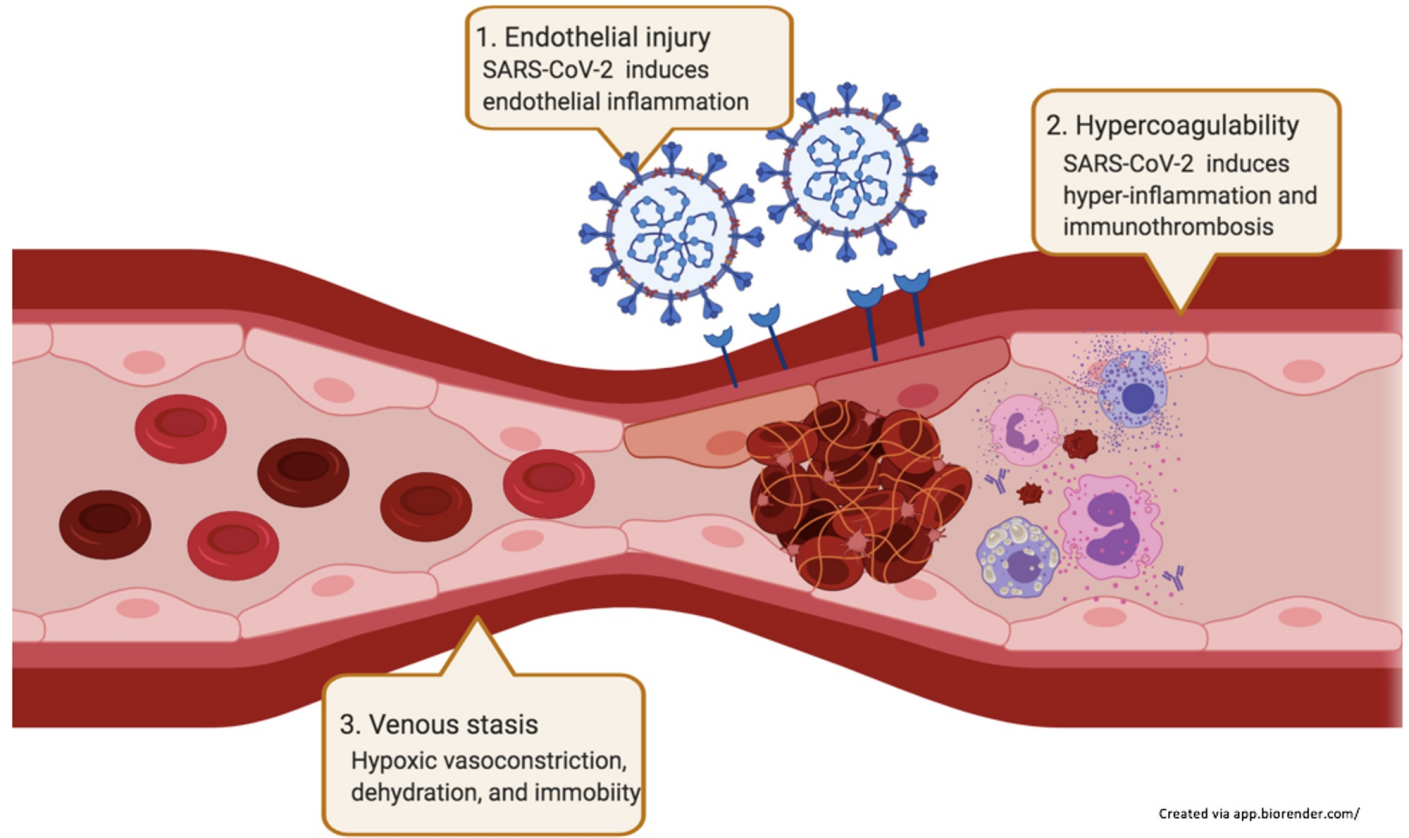
Possible mechanisms of VTE in COVID-19

The mechanisms underlying PE in COVID-19 have not yet been elucidated. Theoretically, COVID-19 may disturb all dimensions of Virchow’s triad: endothelial injury, hypercoagulability, and venous stasis (figure 3). SARS-CoV-2 can directly injure endothelial cells by binding to its functional ACE-2 receptors. Also, the virus can lead to hypercoagulable states via abnormal activation of immune cells, causing hyper-inflammatory responses, aberrant anticoagulant production, and detrimental immunothrombosis [8]. Hypoxemia from SARS-CoV-2 pneumonia further leads to vasoconstriction and venous stasis, aggravating the pro-thrombotic states. Besides, sick patients are likely to have dehydration and decrease mobility. The occurrence of PE even in mild COVID-19 raises the question of whether we should provide prophylactic anticoagulation use in selected mild COVID-19 cases.

## Conclusions

Mild COVID-19 illness may associate with an increased risk of thrombosis. More research is needed to verify our hypothesis and determine if the development of VTE and PE is associated with worsening COVID-19 outcomes. A study exploring the use of anticoagulants in mild COVID-19 cases with certain risk factors, such as malignancy and immune-mediated diseases, will be worthwhile.
